# Secure and Authenticated Data Communication in Wireless Sensor Networks

**DOI:** 10.3390/s150819560

**Published:** 2015-08-10

**Authors:** Omar Alfandi, Arne Bochem, Ansgar Kellner, Christian Göge, Dieter Hogrefe

**Affiliations:** 1 College of Technological Innovations, Zayed University, Khalifa City B, P.O. Box 144534 Abu Dhabi, UAE; 2 Institute of Computer Science, Georg-August-Universität Göttingen, Goldschmidtstrasse 7, 37077 Göttingen, Germany; E-Mails: arne.bochem@stud.uni-goettingen.de (A.B.); kellner@cs.uni-goettingen.de (A.K.); christian.goege@stud.uni-goettingen.de (C.G.); hogrefe@cs.uni-goettingen.de (D.H.)

**Keywords:** sensors, WSN, cryptography, security, authentication, PKI, encryption, MAC

## Abstract

Securing communications in wireless sensor networks is increasingly important as the diversity of applications increases. However, even today, it is equally important for the measures employed to be energy efficient. For this reason, this publication analyzes the suitability of various cryptographic primitives for use in WSNs according to various criteria and, finally, describes a modular, PKI-based framework for confidential, authenticated, secure communications in which most suitable primitives can be employed. Due to the limited capabilities of common WSN motes, criteria for the selection of primitives are security, power efficiency and memory requirements. The implementation of the framework and the singular components have been tested and benchmarked in our testbed of IRISmotes.

## 1. Introduction

Wireless sensor networks (WSNs) consist of multiple distributed sensors that are deployed in an area of interest to sense physical phenomena or environmental conditions, such as temperature, vibrations, sounds, *etc*. The sensor motes act autonomously in a distributed fashion without a central coordination entity, so that the network topology can be created on a peer-to-peer basis on demand. Data are transmitted cooperatively through the network by forwarding the data from mote to mote, resulting in a multiple-hop transmission.

Recently, WSNs have become an increasingly important research topic due to the growing demand for monitoring applications in various contexts, in the civilian, as well as the military sector. Moreover, technological progress enabled the mass production of sensors, resulting in decreased manufacturing costs and, thus, less expensive sensors for customers. While sensor motes are getting smaller and less expensive, their functionality is continuously improved.

One of the main characteristics of sensor motes is that they are usually limited by energy constraints due to the fact that most sensors are powered by battery. For that reason, the motes are often equipped with energy-preserving components, which results in low computational, as well as low transmission power. Furthermore, the available memory is rather limited.

Due to the fact that WSNs are often deployed in unattended or even hostile areas, security-related issues have to be considered. Particularly in medical and military applications, data confidentiality, *i.e.*, keeping the data transmitted by sensor motes confidential, is of high relevance. Furthermore, data integrity often needs to be guaranteed so that modifications of data packets, caused intentionally by an attacker or unintentionally by interferences, can be easily identified. Other concerns, such as the injection of falsified data, replaying of old packets and related issues, also need to be taken into account.

A good counter measure to mitigate those security issues is the application of cryptographic measures. However, in the special case of WSNs, the existing security mechanisms cannot be applied directly to the sensor motes due to their resource constraints in terms of computational power and memory limitations. Therefore, often, only light-weight symmetric cryptography algorithms have been applied on sensor networks [1]. Although, for a long time, researchers neglected public key cryptography on sensor motes, recently, its aptitude was shown in a few papers [2,3]. One of the most promising approaches in this area seems to be elliptic curve cryptography (ECC), which offers a high level of security with small key sizes at an acceptable level of performance.

In this paper, a hybrid cryptography approach is employed to build a simple security scheme, which combines the security benefits of a public key infrastructure (PKI) during the handshake, with the power efficiency of symmetric cryptography for the bulk of communication. This security scheme is implemented for TinyOS [4], an open source operating system for sensor motes. The implementation was simulated in TOSSIM [5], the TinyOS simulator, and, subsequently, tested in our test bed of sensor motes. The motes we used are IRIS motes produced by Crossbow, based on an 8 bit microcontroller with 8 KB of RAM and 128 KB of program memory [6].

During the course of developing this security framework, we have considered and analyzed a variety of cryptographic primitives for their suitability. Therefore, a modular architecture has been chosen for the developed framework, which allows the easy replacement of one component with another, so it can be adapted to various circumstances with different requirements.

Specifically, this paper will give a detailed analysis of the performance characteristics of certain block ciphers (Skipjack [7], RC5 [8], AES128 [9], eXtended Tiny Encryption Algorithm (XTEA) [10]), the Spritz [11] stream cipher, block cipher modes and authenticated encryption modes (counter (CTR) [12], Counter with Cipher block chaining Message authentication code (CCM) [13], Synthetic Initialization Vector (SIV) [14], Offset Codebook mode (OCB) [15]) and MAC functions (hash-based message authentication code (HMAC)-SHA1 [16,17], Cipher-based Message Authentication Code (CMAC) [18], SipHash [19], keyed BLAKE2s [20]) on IRIS motes. The results should be applicable to other platforms based on Atmel ATmega128 microcontrollers, such as the popular MICAz and MICA2 motes.

The rest of the paper is organized as follows: In Section 2, related works of the paper are discussed. Afterwards, in Section 3, the security scheme is presented and its foundations are explained in depth. Then, in Section 4, the conducted simulations, as well as the experiments in our test bed are discussed and evaluated. In Section 5, we describe our methodology for comparing various cryptographic primitives, which can be used for the bulk data communication part of the previously described scheme. Next, in Section 6, the results for the comparison are presented. Finally, in Section 7, conclusions are drawn and future work is discussed.

## 2. Related Works

Several cryptographic methods for wireless sensor networks have been studied and evaluated, due to their constrained resources in terms of energy consumption and computational power. Sensor motes are limited in their computational and memory capabilities, so that the well-known traditional cryptographic techniques cannot be simply transferred to WSNs. TinySec [1], introduced as a security framework for WSNs, addresses security in motes where energy and computational power impose significant limitations on the approaches available for securing such networks. It supports the use of symmetric cryptography, namely block ciphers in cipher block chaining (CBC) mode, to secure sensor mote communications, but it was never ported to TinyOS 2.x, the latest TinyOS version. Recently, there has been a change in the research community from symmetric cryptography to public key cryptography, which has been traditionally considered as too expensive on sensor motes. One main area of interest in the area of public key cryptography is elliptic curve cryptography (ECC) [21]. The advantage of ECC in comparison to other public key approaches, such as RSA, is its mathematical foundation: while the best algorithm that solves the integer factorization is a sub-exponential problem, the best algorithm that solves the ECC discrete logarithm problem is exponential. For that reason, ECC is faster, but at the same time, it can reach equivalent security with smaller keys. The benefit of smaller keys is that they need less processing time, less storage, less bandwidth and, therefore, less energy, which is ideal for energy-constrained sensor motes. As a conclusion, ECC offers an alternative to both symmetric cryptographic systems and more heavy-weight algorithms, such as RSA. Wander *et al.* [2] have previously shown that the use of ECC on wireless sensor motes can be viable. As a result, ECC is likely to play an important role for public key cryptography in WSNs in the future.

As far as implementation goes, TinyECC [22] is a high-speed implementation with real-world security. According to a recent study, if reasonable implementation security is required, it is still the fastest available ECC implementation for AVR-based platforms, such as IRIS and MICAz motes [23].

Since then, various proposals for both pure public key cryptography-based communication systems and hybrid schemes, using both symmetric and asymmetric methods, have been introduced. For example: Pugliese, M. and Santucci, F. [24] discuss in their paper a novel hybrid cryptographic scheme for the generation of pairwise network topology authenticated keys (TAK) in WSNs, based on vector algebra in GF(q). For the ciphering and authentication model, symmetric cryptography is used, while the key generation model is drawn on asymmetric cryptography [25].

Investigations of the efficiency of different cryptographic primitives for their use on wireless sensor networks have been conducted before. A survey of such investigations has been conducted by Roman *et al.* [26]. Most of the surveyed works make use of specialized hardware and asymmetric cryptography, but software implementations of symmetric ciphers are also briefly discussed.

In the aforementioned TinySec framework, which provided a cryptographic communication layer for TinyOS 1.0 using symmetric cryptography, only a single key, and thus, cipher instance, was used, which means that initialization costs and key schedule storage costs were not evaluated in detail [1].

A survey on block ciphers for their use in wireless sensor networks was performed by Law *et al.* [27]. This work concentrated on the use of Smart Dust, EYESnode and Intel mote sensor motes, all of which have rather different performance characteristics than IRIS and similar motes. No analysis of the message authentication code algorithms was performed.

Ganesan *et al.* [28] have also done an analysis on the performance of different cryptographic algorithms on different CPUs used in wireless sensor motes. However, neither the memory used by an instantiated cipher is considered, nor are MAC algorithms or block cipher modes analyzed.

However, at this time, no detailed analysis of OCB [15] and SIV [14] performance on WSN motes has been performed. Additionally, the new SipHash [19] and BLAKE2s [20] functions are similarly unexplored territory.

## 3. Scheme Design

An inherent problem of security schemes in WSNs relying solely on symmetric cryptography is their vulnerability against attackers that gain access to the keys stored on one of the sensor motes. Besides, due to memory constraints and other efficiency reasons, only a limited number of keys can be pre-distributed in WSN scenarios. As a consequence, already a low number of compromised motes allows an attacker to gain access to big parts of transmitted information. Tamper-resistant hardware would provide a way to mitigate this problem, but this is generally too expensive.

### 3.1. Basic Considerations

In this paper, we expand on a previously presented, simple PKI-based scheme for WSNs [29], which is able to prevent this sort of attack. The scheme provides basic security requirements, such as secrecy, authorization of nodes and integrity of messages tailored to the limited resources of the sensor motes. Moreover, an additional mechanism to detect replay attacks is implemented.

The basic idea of the PKI-based scheme is that each sensor mote is assigned a pair of public and private keys, which is then used to derive a symmetric key that can be used for faster symmetric cryptographic methods. If the key for the symmetric cryptography depends on information exchanged by both parties, encrypted towards both parties’ public keys, a certain degree of forward secrecy can be achieved. The scheme elliptic curve integrated encryption scheme (ECIES) [21] is used for the encryption in this process. As a result, compromising a single mote is not sufficient to decrypt the data that are transmitted from or to the mote.

To provide the authorization of sensor motes, a central authority (CA) signs the public key and the network address of each mote. In the next step, the CA’s public key, the mote’s key pair and its address, as well as the CA’s signature are pre-distributed to each mote at compilation time. This enables each mote to verify that other motes’ public keys are authorized to participate in the WSN.

Integrity protection can be provided by the scheme in two ways: using the elliptic curve digital signature algorithm (ECDSA) [21] and using the hash-based message authentication code (HMAC). While ECDSA signatures can be used from the start to provide integrity, HMAC can be used after the symmetric keys are established. The latter approach is however less expensive in terms of computational costs, so that it should be preferred to save the motes’ energy.

Further measures have to be implemented to prevent attackers from replaying captured packets to motes. During the handshake, which is used to establish a connection between two motes, both parties have to ensure that their communication partner is alive and not just replaying old packets, while ignoring their half of the handshake. As mentioned before, the symmetric key should be generated using data contributed by both motes. If one of the motes is simply replaying packets, it will be unable to correctly determine the negotiated key. To ensure that such cases are detected, packets are authenticated using HMACs calculated by using the key negotiated during the handshake. As a block cipher in counter mode [12] is used for the symmetric encryption, protection against replay attacks can be implemented easily by discarding packets with a lower counter value than that of the last accepted packet and adding an HMAC over the encrypted message and counter.

For the symmetric cipher, the tiny encryption algorithm extensions (XTEA) [10] block cipher was chosen for the original implementation, as the algorithm requires basically no key setup or state, so that the memory requirements are just 16 bytes to store the key. At the same time, the computational complexity is not too high either, and it can even be reduced by lowering the number of rounds. However, decreasing the number of rounds results in a decreased level of security. Currently, the best known attack on the algorithm is an attack on 27 of the full 64 rounds by Ko *et al.*, which has a time complexity of 2^115.15^, requiring 2^20.5^ chosen plaintexts [30]. Considering these properties, it still provides reasonably good security in a practical sense, while having favorable properties with respect to use on sensor motes.

Other ciphers under consideration at this state were Skipjack and RC5, which have been used in TinySec [1], but both require more than 100 bytes of state per instance, which was considered problematic, since each connection would be using a different key and, thus, state.

### 3.2. Scheme Description

After start-up, the sensor motes begin broadcasting their certificates. Each broadcast certificate includes the mote’s address, its public ECC key and a signature provided by the CA. To avoid too many concurrent broadcasts, which could not be handled at the same time by receiving nodes, a small random delay is added. To provide resilience in the case of packet loss, during the handshake, packets are presented a predefined number of times if no response has been received.

To save some bandwidth, the certificate broadcast frequency is gradually lowered over time. This can be justified by the assumption that more motes will try to establish a connection at the beginning. Other motes are still able to join the network later, but not as quickly as in the initial setup phase.

Due to the memory limitations of the sensor motes, the number of stored certificates and public keys, as well as the number of current connections and handshakes are limited. Using multiple hops, it should generally still be possible to find a path from any mote within the network to any other. To counteract packet loss, packets sent during handshakes are retransmitted a number of times. Timeouts are used to mitigate incomplete handshakes that cannot be finalized.

When a certificate is received, a mote checks the obtained signature, and if it is valid, the public key and the mote’s address from the certificate are added to its list of known public keys. From this point on, a secured connection can be established between both nodes. Depending on the desired behavior, a handshake can be triggered automatically or on demand, *i.e.*, when the application tries to send data to the mote. The handshake is inspired by the transport layer security (TLS) handshake, but highly simplified [31].

To establish a connection, a mote can send a key exchange offer to another mote from which it has received a public key. The setup of a connection is implemented as follows: at the beginning, 128 bits of random data are generated and stored. These random data are then encrypted using ECIES towards the target mote’s public key. The resulting message will be signed using ECDSA, using the sending mote’s private key. Though ECIES already provides an HMAC over the message, it is not really useful, since this HMAC does not provide sender authentication. Instead ECDSA is used to provide sender authentication, while the HMAC is neither calculated nor sent.

**Figure 1:**
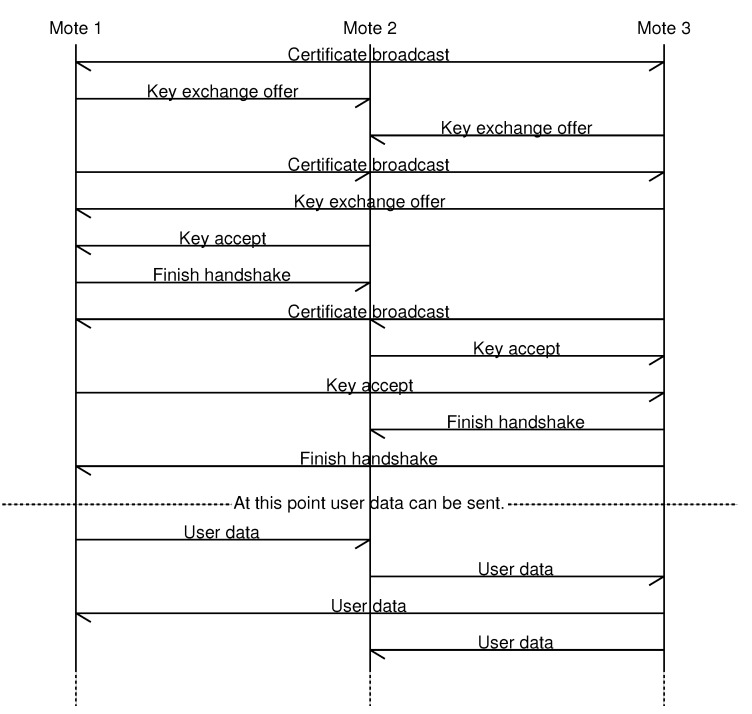
Example handshakes between three motes.

Upon receiving a key exchange offer, the receiving mote will first check that it still has a free connection and handshake slots. If this is not the case, an error message is sent, so that the mote that initiated the key exchange offer can immediately drop the connection attempt and try it again with a different mote. The message sequence chart in Figure 1 shows an example of the handshakes between three motes.

Error packets contain an eight-bit error code and include information about the packet they were sent in response to, as well as an ECDSA signature of the sending mote. If the error packet was sent in response to a user data packet within the context of an established connection, an HMAC can be substituted for the ECDSA signature.

After ensuring that there are still enough resources to accept a connection, the mote that received the key exchange offer will look up the sender’s public key. If it is unavailable, it can either wait for it to be broadcast or it can send an error packet with an error code indicating that the sender’s public key is unknown. On receiving the error packet, the sender will start broadcasting its public key again. If the public key is available, the signature can be checked. If the signature is valid, the received ECIES message will be decrypted and a further 128 bits of random data will be generated and stored. This new chunk of random data will be encrypted using ECIES with the public key of the mote that sent the key exchange offer.

The received 128 bits of random data and the newly generated 128 bits will be XORed and used as input for the ANSI-X9.63 key derivation function [21] to produce two 128-bit keys, one for use in the symmetric encryption within the established connection and the other for use in the HMAC calculation.

*K_xtea_*||*K_hmac_* = *KDF*(*rand_keo_* ⊕ *rand_accept_*)

After deriving the keys, the HMAC of the new ECIES message will be calculated, and a packet containing the ECIES message and the HMAC will be sent to the original mote to announce the acceptance of the key exchange offer.

Upon receipt of the accept packet, the ECIES message will be decrypted, and the contained 128 bits of random data will be used with the original 128 bits to derive keys as specified before. Using the newly derived key, the HMAC of the ECIES message will be verified. If it is valid, a final packet will be sent to the other mote and the connection is marked as “established”. The final packet contains the HMAC of the string “connection key is OK”. This is sufficient to demonstrate to the other mote that no replay attack occurred and the handshake was successfully completed.

Upon the reception of the final packet, the HMAC of the known string will be calculated and checked. If it is valid, the connection will be marked as established.

From this point on, user data can be securely transmitted. The sender of the key exchange offer will initialize its block counter for the block cipher counter mode with zero, while the receiver will initialize it as 2^63^. It is assumed that the motes will not send enough data for the counters to overlap. User data packets contain the length of user data, the block counter that is required for decryption and the user data itself (payload), which is encrypted using XTEA in counter mode. An HMAC is added to the packet to provide integrity protection.

## 4. Evaluation

To verify the practicability of the scheme, a TinyOS implementation was written and tested. The implementation was first tested in TOSSIM, the simulator included in TinyOS, and subsequently, the implementation was tested in a small-scale test bed of IRIS sensor motes. For the implementation, TinyECC was chosen to provide ECC and some cryptographic utility functions [22], as it is an implementation with security sufficient for the real world, as well as comparatively high speed [23] on AVR platforms. Implementations for the XTEA block cipher, a cryptographic random number generator and counter block cipher mode in nesC [32], have been written from scratch. Since a block cipher had to be written anyway, the Counter mode Deterministic Random Byte Generator (CTR_DRBG) [33], has been chosen.

The scheme was implemented as a wrapper around the AMSend and Receive interfaces of TinyOS, with a small number of additional commands and events in a new interface called CryptoLayer. The new interface enables the user to query information about currently connected and known motes, as well as explicitly create and drop connections.

In preparation of flashing the motes, first, a pair of ECC keys will be generated, to be used as keys of a central authority. The private key will be used to sign the public keys and addresses of motes, while the public key will be kept distributed. Then, at compilation time, cryptographic keys for the motes are generated. The mote’s public key and its address are then signed by the central authority. This signature, the CA’s public key and an initial chunk of entropy to seed the random number generator are all also compiled directly into the mote.

The sizes of different packet types used in this implementation are shown in Table 1.

**Table 1:** The sizes of different packet types used in this implementation.

Packet Type	Size (in Bytes)
Certificate	62 B
Key exchange offer	78 B
Accept	58 B
Finish	21 B
User data	≥30 B
Error	52 B

The headers of the underlying AMPacket format add some additional overhead, the exact length of which is platform dependent, but usually in the range of 5–12 bytes.

During development, this implementation was regularly tested using TOSSIM.

### 4.1. TOSSIM

TOSSIM allows the simulation of TinyOS-based sensor motes, directly using the implemented TinyOS application. A Python 2.5 script is used to specify the simulated network’s behavior, such as the number of used motes, the motes’ boot times and their signal strength.

**Figure 2:**
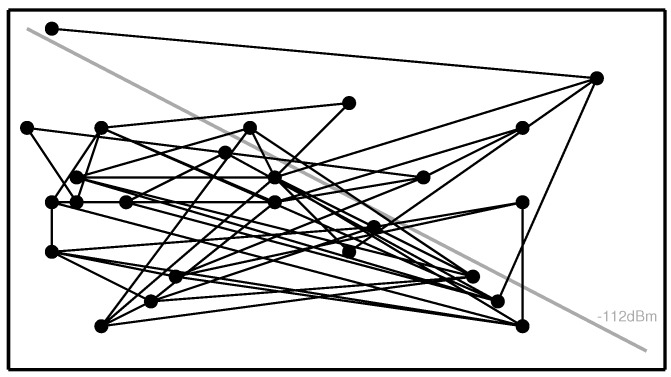
Example network topology in a 25-mote TOSSIM simulation run.

The following scenario has been implemented for testing the security scheme in TOSSIM: in multiple simulation runs, 100 motes are randomly deployed on a 25 m × 50 m grid. The signal strength between two motes is set to be proportional to the Euclidean distance between them, with a gain of 0 dBm at a minimum distance to −112 dBm at maximum distance within the grid, as shown in Figure 2.

The number of concurrently possible connections that can be established with each mote is limited to 10, to imitate the constrained memory conditions on actual motes. Once the motes have established the connections, they start to send user data packets containing the string “user data packet” to their peers at a regular interval of 5 s.

During the simulation, each mote uses the debugging facilities function to output detailed information about its current state and activities, such as the receiving and the sending of packets, the verification of signatures, *etc*. The output is collected in a log file, which is afterwards analyzed to determine how well the simulated WSN is connected at various points in time. In the context of the simulation, a pair of motes is said to be connected, as soon as the first user data packet has been transmitted between them. The connectivity of the whole network is the number of motes within the greatest connected graph of motes at that point in time.

In the following table, the average number of established connections, over multiple simulation runs, at the point in time of achieving a given connectivity is shown in Table 2.

**Table 2:** The average number of established connections, over multiple simulation runs, at the point in time of achieving a given connectivity.

Connectivity	Average Total Connections	SD
≥50% of motes	218	10
≥95% of motes	732	49
=100% of motes	878	29

**Figure 3:**
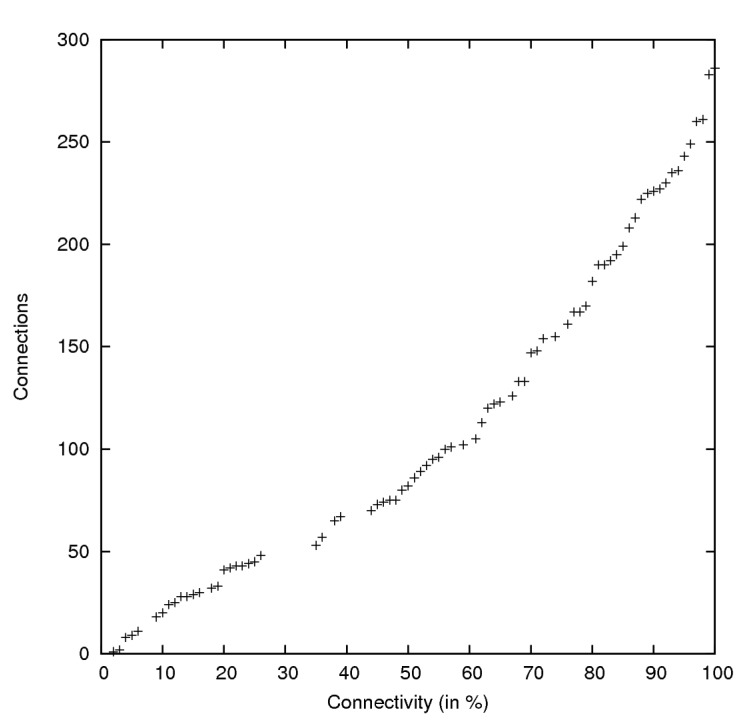
Plot of connections by connectivity of one simulation run.

The simulation results show that in this scenario with 100 simulated sensor motes, 10 connections per mote, on average even less, are sufficient to form a fully-connected network. A plot of the connections by connectivity for one simulation run can be seen in Figure 3.

After getting satisfactory results in the TOSSIM simulation, the implementation was tested in a small-scale test bed of IRIS motes.

### 4.2. Test Bed

**Figure 4:**
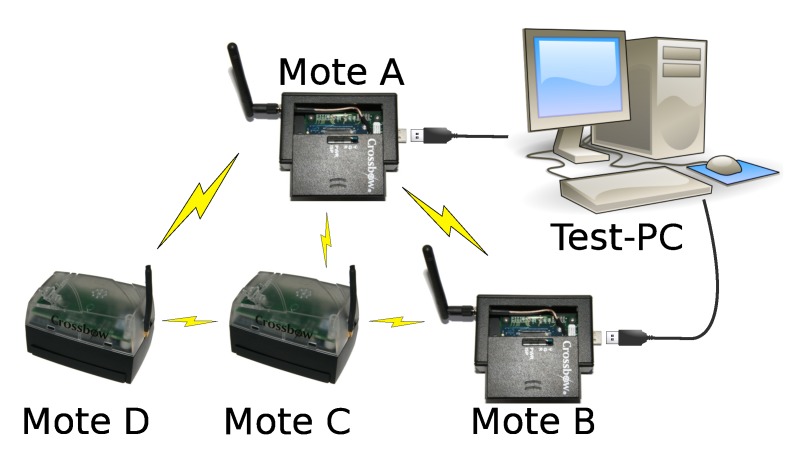
Configuration of the four-mote test bed.

A small experimental setup, consisting of four IRIS sensor motes, has been used to test the implementation in our test bed. A diagram of the setup can be seen in Figure 4. During testing, two of those motes stayed connected to a PC via USB. This connection was used to gather information about the operations within the WSN, as the two connected motes are outputting information about the current state and the received packets by means of the TinyOS printf library. The output was gathered by the PrintfClient TinyOS tool and logged to a file for future analysis.

After generating key material and flashing all four motes, all were switched on/reset simultaneously. The motes correctly exchanged handshakes and successfully transmitted user data messages with a seven octet payload.

## 5. Cryptographic Primitive

To further optimize the performance characteristics of the bulk data transfer after the handshake, we have analyzed alternatives to HMAC-SHA1 and CTR-XTEA, which were originally employed within our scheme.

In this section, the rationale for choosing which cryptographic primitive to analyze, as well as the methodology used for the analysis and measurements are described.

### 5.1. Selection of the Primitive

First, it is necessary to decide on an appropriate selection of the cryptographic primitives, which may be useful alternatives to those currently in use.

#### 5.1.1. Block Ciphers

We have selected four block ciphers: Skipjack, RC5, AES-128 and XTEA. RC5 and Skipjack are selected as they have been successfully used in secure communication systems for WSNs, and thus, implementations are readily available [1,8]. It should be noted that the implementation of RC5 has been extended from allowing only 64-bit keys to allowing 128-bit keys, so as to be in line with today’s security requirements [8,34]. Additionally, the number of rounds has been increased in response to cryptanalytical attacks on regular full-round RC5 [35].

The Skipjack cipher is the only one in the comparison with a maximum key length of less than 128 bits, namely 80 bits. Additionally, the fact that the currently best known cryptanalysis of it breaks 31 of 32 rounds requiring 2^34^ chosen plaintexts with a time complexity of 2^78^ leads to doubts regarding its security. However, due to easily being ported from TinySec to TinyOS 2.x, it has still been included in the comparison.

The XTEA cipher is included, because it is the cipher originally used in the scheme [29]. The original reason for the choice was low memory use, as no key schedule has to be stored, which is useful in an environment with highly limited RAM (8192 byte), when key the schedule has to be stored for each communication partner to avoid repeated reinitialization.

AES128 is the last block cipher included. It provides state-of-the-art security and was designed to have good performance on a variety of platforms [27].

#### 5.1.2. Stream Ciphers

Besides block ciphers, Spritz [11], a modern stream cipher, the design of which is intended to be similar to that of RC4, is included in our comparison. Its cipher state is, with 256 bytes, the biggest of all ciphers, but to emulate the use of a block cipher in CTR mode, we are employing Spritz with IVs, as specified by Rivest and Schuldt. This means that for each packet, the cipher has to be reinitialized with the new IV or sequence number; the cipher state has to be reinitialized, and the memory issue becomes moot, since the state cannot be cached anyway.

#### 5.1.3. Block Cipher Modes

With regard to block cipher modes, which allow the use of block ciphers with data of a size other than the block size of the block cipher, we have chosen four candidates: the first, counter (CTR) mode, is simple to implement and useful as a baseline when comparing performance to other, more complex approaches.

The other selected block cipher modes are modes that provide authenticated encryption instead of encryption only. It should be noted that one possible candidate has been excluded from the comparison, namely Galois/counter mode (GCM). A rough estimate of the cycles required to perform 128-bit multiplication in the GF (2^128^) polynomial field on the targeted eight-bit microcontroller platform indicated that it would suffer from excessive computational overhead [36].

The second selected block cipher mode is CCM (counter with CBC-MAC) mode. It is based on the CTR mode and the CBC-MAC authentication mode, providing both confidentiality and authentication by appending a message authentication code (MAC) to the message. It also allows for the authentication of additional data outside the encrypted region. Only a single 128-bit key is required to use this mode [13].

The third selected mode is OCB (offset codebook) mode. It has been designed as an authenticated encryption mode with minimal overhead. Only a single 128-bit key is required to use this mode [15].

The last mode included is the SIV (synthetic initialization vector) mode. Like the two previous block cipher modes, it provides authenticated encryption. However, it requires a 256-bit key or, rather, separate keys for encryption and authentication. At the same time, it is different from the other modes in that it is not necessary to provide an initialization vector [14].

Further details about the properties of the different modes will be given in the Results Section, giving special consideration to their use in WSNs.

#### 5.1.4. Message Authentication Codes

The first included MAC algorithm is the keyed hash message authentication code (HMAC), which was originally used in the implementation. It was used as it is readily available, due to being implemented in TinyECC, which implemented it as a part of its ECIES implementation. As HMAC here is used with the SHA-1 hash function, this is included in the analysis for reference purposes [16,17,21,22].

The second included algorithm is CMAC, which is a modification to CBC-MAC with improved security properties [18].

Thirdly, SipHash, or more specifically, SipHash-2-4, has been included. SipHash is a relatively new class of keyed hash functions. It was designed by Jean-Philippe Aumasson and Daniel J. Bernstein in 2012, with the goal to be cryptographically secure, while also providing high performance for short messages [19].

Finally, the speed-optimized BLAKE2s hash function, which is based on the SHA-3 finalist BLAKE and optimized for platforms with eight-bit to 32-bit processors and has a built-in MAC mode, has been also added to the comparison, with a digest size of 160 bits [20].

### 5.2. Static Analysis

After choosing the cryptographic primitive, a static analysis is performed. To do this, a simple TinyOS application using a given primitive is compiled for the IRIS platform, while instructing the compiler to keep the generated assembly code. Care is taken to disable inlining of the functions of interest. These functions are then extracted, and conditional branching instructions are annotated to facilitate quasi-execution by a simple parser, which counts the number of cycles required to execute the instructions. In the case of instructions with possibly varying execution times, the worst case is assumed.

With most analyzed functions, the annotation of conditional branching instructions worked without any issues. However, in the case of RC5, the number of times a branch is taken can depend on the input data, due to data-dependent shift operations. The Atmel ATmega128 microcontroller only provides instructions that shift a register by a single bit. Hence, multi-bit shifts are implemented as loops.

In this case, usually the worst case is assumed when annotating the branches. It should be noted that this data dependency of execution time might be problematic from a security point of view, as it might enable a crafty attacker to construct a timing attack.

For SipHash, only the round function is analyzed, as most of the time spent processing the input will be spent there. The total time spent on SipHash-C-Dfor a message of Neight-byte blocks can be then computed by means of the following formula, plus a small overhead: (*N* × *C* + *D*) × *round*_*function*.

In the case of SHA-1, the analysis is performed under the assumption that the update function is called only with 64 byte-long inputs, which corresponds to the internal block size of SHA-1. For all other cases, the block size of the (underlying) cipher is assumed. The key length is always assumed to be 128 bits.

### 5.3. Benchmark

To measure the performance of the various cryptographic primitive in the real-world scenario, a small TinyOS application has been written. This application contains a loop of 100 iterations, where each iteration executes the function to be measured. The runtime of this loop is measured. Afterwards, a message is sent to the PC that the mote is attached to, containing the runtime. Once the message is sent, the function containing the loop is called again. For the first 100 times, the loop is executed without calling the actual cryptographic function, to measure the overhead of the measurement methodology. Multiple runtimes of the 100 iteration loops are then averaged, the overhead subtracted and the result divided by 100 to determine the average runtime of a single iteration.

When benchmarking the CTR, CCM, SIV and OCB modes, as well as CMAC, SipHash, HMAC, BLAKE2s and SHA-1, these measurements are run for message sizes starting with four bytes up to 128 bytes, increasing in steps of four bytes. Since the maximum message data length that can be transferred via radio on IRIS motes running TinyOS is 114 bytes (127 according to IEEE 802.15.4, with a 13-byte header), this covers the complete practical range of message sizes.

## 6. Measurements

In this section, we will present the results of our analysis and measurements.

### 6.1. Static Analysis

In Table 3, we present the approximate cycle counts and other properties of the block ciphers chosen for analysis. SipHash, although it is not a block cipher, has also been included in the table. Information on how to read the SipHash entry is provided in the last part of this section.

**Table 3:** Cycles required per operation/block. BS, block size.

Cipher	BS	Encrypt	Setup	Total min	Memory
Skipjack	64 bits	2452	2860	*5563*	128 bytes
AES	128 bits	9677	3292	12,969	176 bytes
XTEA	64 bits	20,070	*91*	20,161	*16* bytes
RC5	64 bits	11,073	80,258	91,331	104 bytes
Spritz (stream cipher)	for 128 bits	*1117*	74,997	76,114	256 bytes
SipHash (short MAC)	64 bits	756	1512	2268	-

Since, in a hybrid PKI-based communication scheme, a unique session key is negotiated for each connection between two sensor motes and RAM on IRIS motes is limited (8 KB), a trade off has to be made when choosing a cipher. Either the key schedule can be cached for future use or it can be discarded after finishing a batch of encryption operations, such as during encryption using a block cipher mode, so it has to be reinitialized the next time data to or from this peer has to be encrypted or decrypted.

The RAM column in the table specifies the memory required to store the key schedule, while the setup column gives the number of cycles required to perform the key setup. The “total min” column gives the minimum number of cycles that have to be spent to encrypt one single block of the size given under block size (BS).

Even though XTEA requires the least memory, including the marginal key setup costs, it is the second slowest cipher. RC5 has a very slow key setup and the encrypt function is the second slowest in the comparison. Skipjack is the fastest cipher, but due to the smaller key size and low security margins, it should probably not be used, unless performance is of higher importance than security in a given use case. AES seems to offer the best trade-off between speed and security, even when mitigating its higher memory requirements by not caching the key schedule.

For AES, it also needs to be noted that, due to the higher block size, twice as much data can be encrypted with a single call to the encrypt function, leading to comparatively higher performance for longer messages, which takes less than twice as many cycles as Skipjack.

In the case of the Spritz stream cipher, a 128-bit block of data was encrypted very quickly, but the key setup is the second slowest in the whole comparison. This indicates that it might become worthwhile to use for long message, where the higher key setup time is offset by less cycles per byte as far as throughput is concerned.

To generate an SHA-1 digest, three functions are necessary: reset, update and digest. The reset function initializes the context structure used by the implementation. The update functions adds new data to be hashed and, once the internal block size of 512 bits is reached, calls the block processing function. The digest function is called once all data have been added. It performs padding as necessary and calculates the final hash value, again calling the block processing function. For this static analysis, we have assumed that only data sized as a multiple of the internal block size is added. Otherwise, depending on the exact block size, the update function might not call the block processing function, while the digest function may call it twice to process the remaining data. The difference in overall cycles should be small enough to be omitted, as most of the time is spent in the block processing function. The minimum total cycles for calculating an SHA-1 hash on IRIS are 131,685. A breakdown of cycles is given in Table 4. The block processing function is only given for reference purposes and already included in the cycle counts of the other functions.

For the HMAC, CMAC, CTR, CCM, SIV and OCB functions, the main point of interest is the number of calls to cipher and digestfunctions, as the number of cycles spent in their actual functions is rather low (e.g., HMAC spends approximately 670 cycles outside of calls to the SHA1 module for a 512-bit block).

The performance of SHA-1 is thus: generating the HMAC of a 512-bit block of data will take roughly 340,000 cycles, while generating the HMAC of 448 or less bits of data will take about 275,000 cycles.

**Table 4:** Cycles for SHA-1 functions, assuming full 512-bit blocks.

Function	Cycles
Skipjack	100
process block (PB)	62,175
update	1820 + PB
digest	5415 + PB
min total	131,685

For CMAC and CCM, the only approved cipher is AES, according to the corresponding NIST Special Publications, so the required cycles can be looked up in Table 3. CMAC requires roughly *init* + (1 + *blocks* × *encrypt*) cycles, which produces a 128-bit MAC. For CCM, approximately *init* + (2 + 2 × *blocks* × *encrypt*) cycles are required to encrypt *blocks* 128-bit blocks of data and to generate a 128-bit MAC. For each block of additional, associated data to be included in the MAC, one additional encrypt operation is needed.

For CTR mode, approximately *init* + *blocks* × *encrypt* cycles are required. If one were to reproduce the result of CCM using CTR and CMAC, the required cycles would end up as 2 × *init* + (2 + 2 × *blocks*) × *encrypt* with one additional block of encryption being performed to encrypt the MAC.

SIV mode uses CMAC to generate a MAC, which is also used as a nonce for encryption, which is done practically the same way as CTR mode. For that reason, the required cycles should be approximately the same as CTR mode used together with CMAC. However, if any associated plaintext is used in addition to regular plaintext, the overhead grows due to multiple calls to CMAC.

For OCB mode, which is a “one pass” authenticated encryption mode, approximately *init* + (3 + *blocks*) × *encrypt* cycles are required to produce both ciphertext and a 12-bit MAC.

In the case of SipHash-2-4, Table 3, the “encrypt” column gives the amount of cycles to run the round function two times (C = 2), while the setup column gives the amount of cycles to run the round function four times (D = 4). This representation has been chosen, as for every SipHash call, four calls to the round function are made as a constant overhead, like the initialization step of a block cipher, while two calls are made per input block, making it similar to the encrypt function of a block cipher. Overall, it can be seen that SipHash is the fastest performing function in the table, according to cycle count, while also not requiring any static memory to store a key schedule or similar. Some additional notes on usage will be given later in this paper.

The decryption and verification of the MACs have the same complexity with respect to required cryptographic operations.

### 6.2. Benchmark

Having done a static analysis based on the assembly generated from the cipher source code, benchmarks are performed to verify that the results from the previous section still hold, when running on actual sensor motes.

**Figure 5:**
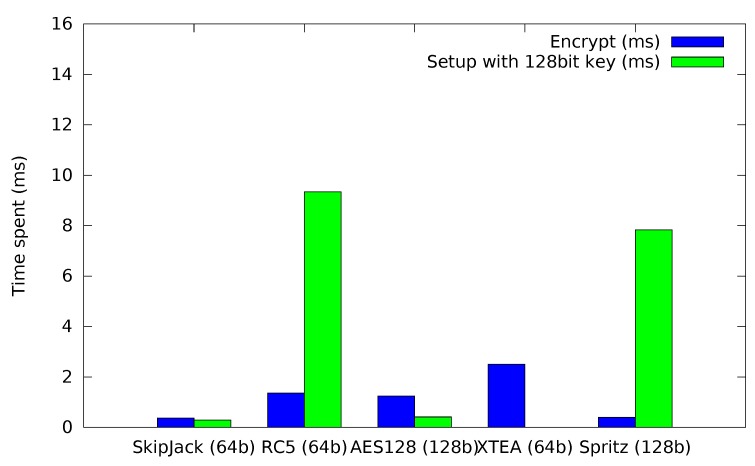
Time required for cipher setup (128-bit key) and encryption.

First, the block ciphers themselves are compared directly, *i.e.*, without any cipher modes. The time required to encrypt a single block of data or to perform the cipher initialization with a 128-bit key can be seen in Figure 5. As expected, Skipjack and AES128 perform quite well. For the XTEA cipher, the time required for initialization is too small to be properly displayed on the graph. Spritz mirrors the expected performance from the cycle analysis.

**Figure 6:**
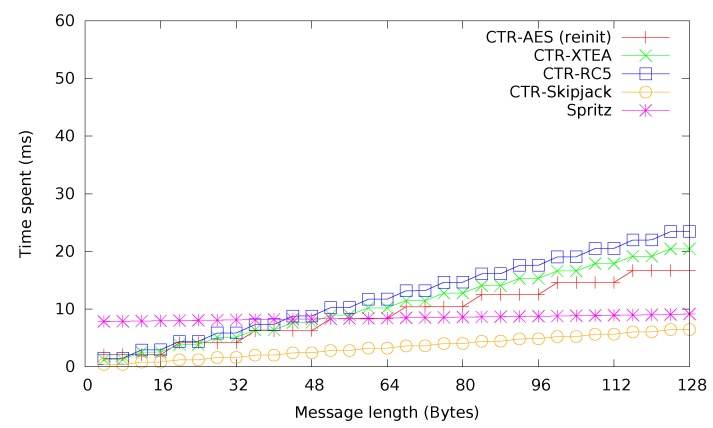
Ciphers in counter (CTR) mode.

Next, the ciphers are compared when used in CTR mode, over messages sized from four bytes up to 128 bytes, basically covering the effective useful range for radio communication on IRIS and similar sensor motes. The results of this comparison can be seen in Figure 6. Again, Skipjack and AES perform best. While for smaller messages, XTEA with 32 rounds performs better than RC5. Starting with a message length of approximately 48 bytes, for pure encryption, the Spritz stream cipher becomes a viable option, its nearly constant performance providing noticeable benefits for long messages.

**Figure 7:**
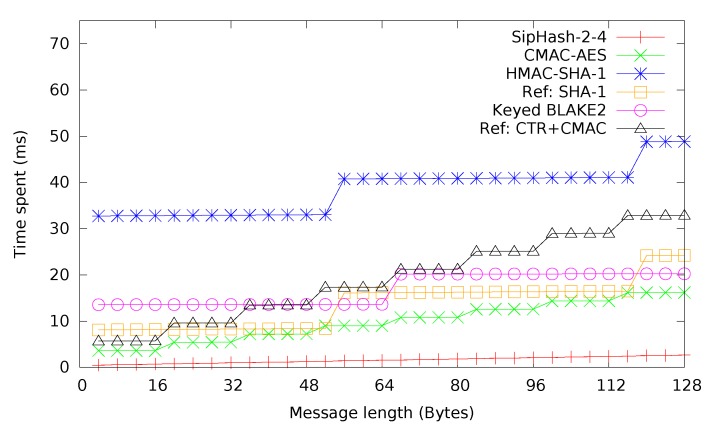
MACs with CTR + CMAC(AES) and SHA-1 for reference.

SipHash-2-4, HMAC-SHA-1, BLAKE2s and CMAC are compared in Figure 7. For reference purposes, raw SHA-1 and CTR + CMAC have been included, too. As expected, HMAC-SHA-1 takes about four-times as much time as SHA-1 for small messages, while the difference becomes smaller as message sizes grow. CMAC and CTR + CMAC both perform much better than HMAC-SHA-1. SipHash-2-4 has the best performance by far, as was to be expected according to Table 3. SHA-1 mostly performs better than BLAKE2s, but since BLAKE2s allows keying, for direct use as a MAC, it should instead be compared to HMAC-SHA-1, in which case the comparison is favorable for BLAKE2s.

**Figure 8:**
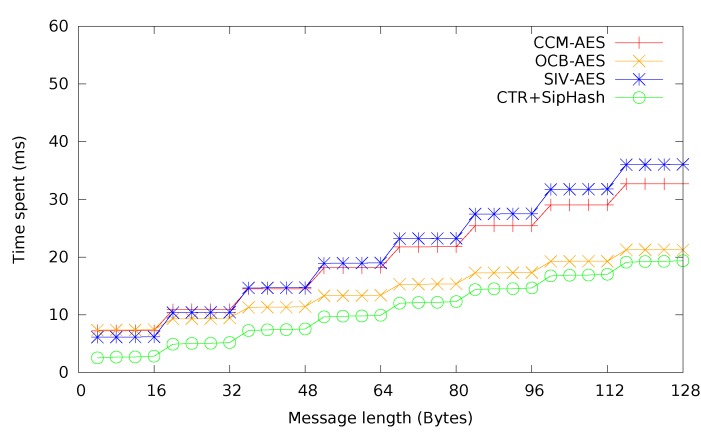
Time required for authenticated encryption with different modes. CCM, counter with cipher block chaining (CBC)-MAC.

In Figure 8, CCM, SIV and OCB mode are compared. Additionally, expected times for CTR + CMAC and CTR + SipHash are provided, to give a comprehensive overview of different authenticated encryption methods. Due to the slow performance of HMAC-SHA-1, CTR + HMAC-SHA-1 has been omitted. As expected, the single-pass OCB mode performed best for most messages sizes, followed by CTR + SipHash, which profits greatly from the high speed of SipHash to lower authentication overhead and is actually even faster than OCB mode for short messages. CCM mode and CTR + CMAC are practically identical, which is not surprising, considering the way CCM mode is constructed. It can be seen that SIV mode has a slight, additional overhead over CCM mode.

**Figure 9:**
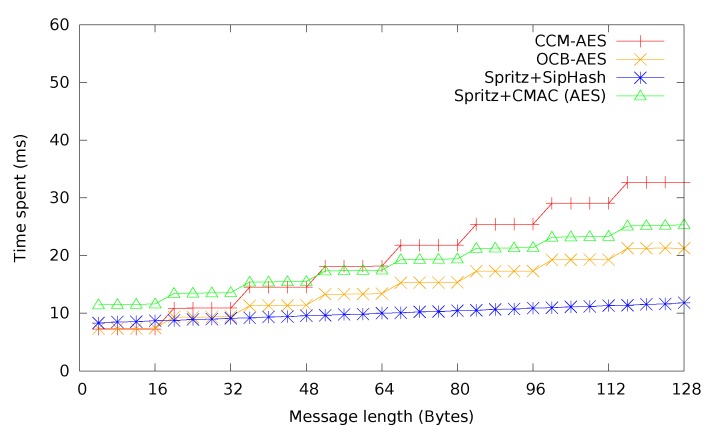
Modes compared with expected Spritz + MAC.

Finally, in Figure 9, CCM and OCB, chosen as the most representative of authenticated encryption modes from the previous comparison in Figure 8, are compared with the estimated time required for either Spritz + CMAC or Spritz + SipHash. Similar to the comparison in Figure 6, Spritz + CMAC becomes a viable option in comparison to CCM mode for messages longer than 48 bytes, while never beating OCB mode. Spritz + SipHash, however, becomes the most performant option already at above 16 bytes, beating even OCB mode. In scenarios where performance is so important that a shorter MAC becomes acceptable, Spritz + SipHash may be a legitimate option for all but the shortest messages. Depending on the chosen primitives, it may also be worthwhile to automatically switch between one, e.g., CCM and Spritz + CMAC, depending on the message length. Of course, this incurs a slight complexity penalty and will increase the code size, which might be undesirable on limited systems, such as WSN motes.

## 7. Conclusions

In this paper, a simple secure PKI-based scheme for WSNs was described and analyzed. It should guarantee the basic security requirements of authentication, confidentiality and integrity during data transmission between sensor motes. The scheme is based on a hybrid cryptographic approach utilizing both public key cryptography, more precisely ECC, during the handshake phase and symmetric key cryptography for the later exchange of user data messages.

As a prerequisite of the presented security scheme, at compilation time, a certificate, which is signed by a CA, is assigned to each mote. Based on the certificates, a key exchange is initiated during the handshake, resulting in two new generated keys that are later used for the symmetric encryption of the user packets and the integrity checking of data packets.

Two experiments were set up, a simulator-based experiment with TOSSIM and a real scenario in a sensor test bed. As the first results of the TOSSIM simulation, it can be highlighted that for the tested simulation scenario with 100 simulated sensors, on average, less than 10 connections are required to obtain a secure, fully-connected network. The results of the experiment show that the motes can correctly complete the handshakes and successfully transmit user data messages.

As mentioned before, when looking only at the block ciphers themselves, AES128 seems to offer the best trade-off between memory consumption, speed and security. It also became obvious that HMAC-SHA-1 should probably be avoided on IRIS and similar WSN motes, as its cycle cost is very high when compared to other MAC algorithms.

For authenticated encryption, OCB mode offers the best performance. However, there are also drawbacks that need to be considered: Firstly, the algorithm for OCB mode is patented in the USA. While a free patent license is granted for many uses, depending on the intended use, additional costs may be incurred due to this. There is another drawback, which may be especially problematic in the case of WSNs, namely the fact that both confidentiality and authentication are completely compromised if a nonce is reused [15]. In the case of WSN crypto systems, implemented in TinyOS, care must be taken with respect to nonce and key generation, otherwise it might be possible for an attacker to restart a mote and reset the PRNGto some known state, which may lead to key and nonce reuse. Furthermore, OCB mode is the only mode in consideration that requires an implementation of the block cipher’s decryption function for decryption. In general, while OCB offers the fastest encryption and MAC generation, it also appears to be the least robust of the analyzed methods.

Performance-wise, Spritz + SipHash is the second best option. In this case, nonce reuse will only compromise confidentiality. However, it should be noted that the MAC generated with SipHash will be shorter than the other candidates, with only 64 bits [19]. While this lowers security margins, it may be an acceptable tradeoff for certain scenarios where it is unlikely that attackers will spend a lot of time on breaking security.

The behavior of CCM mode and CTR + CMAC is basically identical, with CCM mode being slightly faster. If the better security margins of a longer MAC are necessary, CCM mode is a feasible alternative to CTR + SipHash. Having good robustness and security characteristics, while also offering decent performance, it may be the most appropriate choice for security-sensitive fields, such as medical WSNs.

For messages longer than 48 bytes, Spritz + CMAC becomes a viable option and has been found to be faster than CCM mode.

SIV ranked last for performance in the benchmark section. It also is the only one of the authenticated encryption modes that requires a key of double length (256 bits) or, more specifically, two distinct keys for CMAC and CTR mode, respectively. However, it may still be worthwhile to consider for use in WSNs. The fact that it generates nonces from the input, as well as the fact that the only information leaked in the case of nonce reuse, which only happens when identical messages are sent, is that an identical message is being sent, make it much more robust in practical use cases than the other approaches. If sequence numbers or timestamps are included in the messages, the remaining leak can also be mitigated. For use cases with a lower message volume or with higher security requirements, the added robustness may make SIV mode a worthwhile choice.

Overall, we have shown that state-of-the-art authenticated encryption methods offer a variety of options to improve performance and energy efficiency, depending on the required level of security and robustness.

Having shown the general feasibility and scalability of the scheme, in the future, it will be further evaluated in terms of scalability outside of simulations. Additionally, various performance metrics will be analyzed, as well as the impact of using different cryptographic primitives in practical scenarios. Additional work can also been done on enhancing the robustness of the scheme when faced with transmission errors and packet loss.
